# Evaluation of Restrictions on Tobacco Sales to Youth Younger Than 21 Years in Cleveland, Ohio, Area

**DOI:** 10.1001/jamanetworkopen.2022.22987

**Published:** 2022-07-12

**Authors:** Erika Trapl, Stephanie Pike Moore, Catherine Osborn, Neha Gupta, Thomas E. Love, Tyler G. Kinzy, Audrey Kinsella, Scott Frank

**Affiliations:** 1 Prevention Research Center for Healthy Neighborhoods, Case Western Reserve University, Cleveland, Ohio; 2 Case Western Reserve University, Cleveland, Ohio; 3 Population Health Research Institute, The MetroHealth System, Cleveland, Ohio

## Abstract

**Importance:**

Tobacco 21 (T21) policies raise the minimum legal age to purchase tobacco from 18 to 21 years to curb youth access to tobacco products. While some studies have found that T21 is associated with reducing prevalence of youth tobacco use, little is known about the impact it may have on youth of different racial and ethnic identities.

**Objective:**

To evaluate the association of T21 policy with the prevalence of high school youth tobacco use across sex, race, and ethnicity.

**Design, Setting, and Participants:**

This survey study used representative survey data collected from the local biennial Youth Risk Behavior Survey from 2013 to 2017 comparing Cleveland, Ohio (which has a T21 policy), to proximal jurisdictions in the first-ring suburbs in Cuyahoga County (which do not have T21 policies). Within-Cleveland demographic information was also collected for 2013 to 2019. Overall high school youth tobacco use rates were compared between Cleveland and the first-ring suburbs and then examined within Cleveland among Hispanic, non-Hispanic Black, and non-Hispanic White high school students. Percentage data were adjusted to more closely align with local population demographics. Data were analyzed from January to June 2022.

**Exposures:**

T21 was implemented in Cleveland in 2016 and not adopted in proximal jurisdictions or at the state and federal level until at least 1 year later.

**Main Outcomes and Measures:**

The main outcomes were prevalence of past 30-day cigarette, cigar product, or e-cigarette use, measured using geographically representative high school youth survey data from 2013 to 2015 (prelegislation) and 2017 to 2019 (postlegislation) and compared using a difference-in-differences analysis.

**Results:**

The unweighted sample included 12 616 high school students (27.0% [95% CI, 26.9%-28.0%] in 10th grade; 50.9% [95% CI, 50.3%-51.6%] females) participating in 1 or more Youth Risk Behavior Surveys from 2013 to 2019, including 7064 students in Cleveland and 5552 students in the first-ring suburbs. Compared with the first-ring suburbs, Cleveland had a greater proportion of younger students (1623 [28.5%] ninth grade students vs 2179 [34.0%] ninth grade students) and Hispanic students (436 students [1.1%] vs 1433 students [12.6%]) and non-Hispanic Black students (2000 students [53.1%] vs 3971 students [75.1%]). Cigars were the most commonly used tobacco product in Cleveland, with use reported by 6201 students (19.8%) in 2013, 5877 students (21.3%) in 2015, and 5784 students (16.8%) in 2019. Compared with the first-ring suburbs, there was a greater decline in prevalence of use of cigars in Cleveland (β = 0.18 [SE, 0.05]; *P* < .001). The disparity across race, ethnicity, and sex decreased for all current tobacco product use. For example, the maximum difference between demographic subpopulations in current cigarette use was 11.6 (95% CI, 9.5-13.7) percentage points in 2013 between White females (16.1% [95% CI, 11.3%-20.8%]) and Black males (4.5% [95% CI, 3.5%-5.4%]). This maximum difference in current cigarette use decreased significantly to 5.1 (95% CI, 3.5-6.7) percentage points in 2019 between White females (6.9% [95% CI, 3.4%-10.3%]) and Black females (1.8% [95% CI, 0.7%-2.8%]).

**Conclusions and Relevance:**

This survey study found that there was a decline in youth-reported tobacco use across every tobacco product category from 2013 to 2019. This decline changed the trajectory of use among several demographic groups and brought the youth populations with the highest tobacco product use to similar rates of others.

## Key Points

**Question:**

Is the legislation raising the minimum legal age to purchase tobacco to 21 years in Cleveland, Ohio, associated with equitable outcomes among the adolescent population?

**Findings:**

In this survey study including 12 616 high school students from Cleveland and its first-ring suburbs, prevalence of the most common form of current tobacco use among youth, cigars, declined in the postlegislation period, and there was a substantial reduction in the disparities among racial and ethnic populations across all tobacco product use types.

**Meaning:**

These findings suggest that reduction in youth tobacco product use overall and tobacco use disparities may be associated with diminishing tobacco-related health disparities.

## Teaser

This survey study evaluates the associations of Tobacco 21 policy with prevalence in of high school youth tobacco use across sex, race, and ethnicity.

## Introduction

The decline in tobacco use in the US has been a notable public health achievement,^1^ yet the prevalence of tobacco use among youth remains high, at 31.2% among high school students and 12.5% among middle school students.^2^ There are substantial racial, ethnic, and sex disparities in how and what types of tobacco products are used by different adolescent populations,^3,4,5^ which are likely compounded by disparities in tobacco retail density,^6,7,8^ age restrictive sales adherence,^9^ and targeted marketing by the tobacco industry.^10,11,12^ Population-level interventions and policies are recommended to alter population and societal norms^13,14^; however, care must be taken in implementing these strategies so as to not exacerbate existing inequities.^15^

Efforts to increase the minimum legal purchasing age for tobacco products from 18 to 21 years, often referred to as *Tobacco 21* (T21), have gained national traction, with T21 being adopted into federal law in 2019.^16^ T21 is intended to reduce adolescent initiation of tobacco product use by directly reducing access to tobacco products and by reducing access for near-age peers who may supply tobacco products to youths younger than 18 years^17^ and has demonstrated beneficial associations for reducing youth tobacco use overall.^18,19,20^

To date, few studies have examined how T21 has been implemented across communities and subsequently impacted adolescents across race, ethnicity, and sex. One of the first jurisdictions to implement T21 found cigarette use declined among males and females as well as White youth and youth who were members of racial or ethnic minority groups, such as Black and Hispanic youth, but did not explore trends for other tobacco products nor examine for potential disparities in implementation.^21^ In California, T21 had mixed associations with changes in prevalence of cigarette, smokeless tobacco, and e-cigarette use among adolescents with different racial and ethnic backgrounds.^22^

The city of Cleveland, Ohio, implemented its T21 policy in April 2016.^23^ Cleveland has a high prevalence of adult tobacco use,^24,25^ high rates of poverty, and is a minority-majority jurisdiction (ie, more than half of the population identify with ethnic or racial minority groups),^26^ that has consistently been identified as one of the most segregated cities in the US.^27^ These factors allow us to examine policy equitability more rigorously. Cleveland’s legislation increased the minimum legal tobacco purchasing age to 21. The purpose of this study was to both evaluate the association of Cleveland’s T21 policy with the prevalence of cigarette, cigar product, and e-cigarette use across different high school youth populations and the association of the legislation with the disparities among different sex, racial, and ethnic demographic groups. Compared with a proximal jurisdiction with no T21 policy, a significantly greater decline in high school youth tobacco product use for each product was expected. Within Cleveland, implementation of T21 was expected to contribute to reduced disparities across all demographic groups.

## Methods

For this survey study, data collection was approved by the institutional review board at Case Western Reserve University. Consent forms were sent to homes of students in participating schools. Parents or guardians who approved for their student to participate took no action, while parents or guardians who did not want their student to participate informed their school. The day of the survey, students were provided assent information. Student participation was voluntary and anonymous. Student nonparticipation was due to absence on the day of survey administration, parental refusal, or student refusal. This study is reported following the Strengthening the Reporting of Observational Studies in Epidemiology (STROBE) reporting guideline.

### Population

High school–aged youth in Cleveland, Ohio, were the population of interest in this study. Cleveland is nested within Cuyahoga County, Ohio. High school–aged youth from the first-ring suburbs of Cuyahoga County^28^ were chosen as proximal comparator to examine the effectiveness of the policy. The first-ring suburbs are comprised of 19 distinct municipalities that directly surround the city of Cleveland. One municipality within the first-ring suburbs implemented T21 legislation in mid-2017; no other jurisdiction was impacted by state or federal T21 policies until 2019.

### Data Source

Representative survey data collected among high school–aged youth were collected from the Cleveland-Cuyahoga County Youth Risk Behavior Survey (CC-YRBS). These cross-sectional data were used to evaluate tobacco use trend in the prelegislative (ie, 2013 and 2015) and postlegislative (ie, 2017 and 2019) periods. Methods for collecting CC-YRBS survey data are described elsewhere.^29,30,31,32^ Participation in the CC-YRBS was anonymous and voluntary.

Individual responses were weighted for student nonresponse and by grade, sex, race and ethnicity, and geographic region (categorized as Cleveland East, Cleveland West, First Ring East, First Ring West, Outer Ring East, Outer Ring West). For this study, responses for Cleveland East and Cleveland West were combined to represent Cleveland and First Ring East and First Ring West were combined to represent the first-ring suburbs (FRS). As a result, Cleveland’s responses are mutually exclusive from the FRS.

In Cleveland, response rates were 68.1% in 2015, 66.5% in 2015, 69.3% in 2017, and 76.0% in 2019. Response rates for the FRS were 67.0% in 2013, 56.3% in 2015, and 52.1% in 2017; 2019 prevalence estimates were not included for a regional comparison owing to the adoption of T21 in 2019 at the state and county level.

The response rates for FRS in 2015 and 2017 were below 60%, which was the response rate recommended by the Centers for Disease Control and Prevention. However, in 2019, the Centers for Disease Control and Prevention indicated that jurisdictions with 50% to 60% response rates could be weighted if nonresponse bias analyses indicated no significant bias.^33^ For the FRS, the weighted sample percentages by grade, sex, and race and ethnicity were not significantly different from the population, indicating no significant bias.

### Demographic Characteristics

Demographic comparisons were made between Cleveland and FRS. Demographics from the weighted 2013 geographically representative sample were examined by self-identified grade (9th, 10th, 11th, 12th grade), race and ethnicity (Hispanic, non-Hispanic Black [hereafter, *Black*], or non-Hispanic White [hereafter, *White*]), and sex (male or female). Within Cleveland, prevalence in adolescent tobacco product use was compared across race, ethnicity, and sex to create 6 distinct groups of students who self-identified as Black males, Black females, Hispanic males, Hispanic females, White males, and White females.

### Youth Tobacco Use

Prevalence of tobacco product use among high school youth was assessed using self-reported past 30-day use of cigarettes, cigar products, and e-cigarettes. Cigarette use was measured using the question, “During the past 30 days, on how many days did you smoke cigarettes?”^34^ Cigar product use was determined using the question, “During the past 30 days, on how many days did you smoke cigars, cigarillos, little cigars, or flavored cigars such as Black & Milds, Swisher Sweets, or Phillies?”^35^ e-Cigarette use was determined using the question, “During the past 30 days, on how many days did you smoke an electronic vapor product?”^34^ Current use was defined as use of the tobacco product on at least 1 day in the past 30 days. Prevalence of product-specific use was calculated overall for 2013 to 2019, with the exception of e-cigarette use, which was not captured in 2013 survey.

### Statistical Analysis

We used χ^2^ tests to examine differences in demographic groups between Cleveland and FRS. High school youth tobacco use across both samples was compared prelegislation (2013-2015) and postlegislation (2017) using a difference-in-differences (DID) analysis. Estimates for 2019 were excluded owing to the widespread adoption of T21 at the local, state, and federal level. The sample sizes used to compare jurisdictions in Cleveland were 7064 in 2013, 6197 in 2015, and 6397 in 2017, and in FRS, they were 5552 in 2013, 2797 in 2015, and 4233 in 2017. DID models were adjusted for grade level and racial composition based on demographic differences and prior research illuminating differential associations in youth in younger grades.^20,36^

DID was also used to compare each individual demographic group with the demographic group with the highest prevalence of use for each individual product at baseline being considered the reference group. More than 99% of the Cleveland sample self-identified as Black, Hispanic, or White; students identifying in other racial groups (eg, American Indian or Alaska Native, Asian, Native Hawaiian or other Pacific Islander) were excluded from this analysis. As a result, the biennial sample size used to examine within-Cleveland demographic differences was 6022 in 2013, 5402 in 2015, 5638 in 2017, and 5231 in 2019. DID models were similarly adjusted for grade level.

A measurement of disparity was also included but not statistically tested. Our measure of disparity assessed the absolute difference between the demographic group with the highest prevalence estimate and the demographic group with the lowest prevalence estimate for each of the 3 tobacco product use types assessed.

Analyses were calculated using SAS statistical software version 9.4 (SAS Institute). *P* values were 2-sided, and statistical significance was set at *P* = .05. Data were analyzed from January to June 2022.

## Results

### Demographics

The unweighted sample included 12 616 high school students (27.0% [95% CI, 26.9%-28.0%] in 10th grade; 50.9% [95% CI, 50.3%-51.6%] females) participating in 1 or more Youth Risk Behavior Surveys from 2013 to 2019, including 7064 students in Cleveland and 5552 students in the FRS. The weighted sample of Cleveland and the FRS differed by grade and race and ethnicity but not by sex. Cleveland youth were younger, with a greater proportion of 9th graders (2179 students [34.0%]) compared with high school youth in FRS (1623 students [28.5%]) (Table 1). Additionally, Cleveland had a higher proportion of Black (3971 students [75.1%]) and Hispanic (1433 students [12.6%]) youth compared with FRS, which had 2000 Black students (53.1%) and 436 Hispanic students (1.1%). These demographic differences were consistent across data collection years.

**Table 1.** Sample Demographics of High School Youth in Cleveland, Ohio, and First-Ring Suburbs of Cuyahoga County, Ohio, From the 2013 Cleveland-Cuyahoga County High School Youth Risk Behavior Survey

Characteristic	No. (%) [95% CI]^a^		*P* value^b^
	Cleveland (n = 7064)	First-ring suburbs (n = 5552)	
Grade			
9th	2179 (34.0) [32.3-35.7]	1623 (28.5) [26.9-30.1]	<.001
10th	1804 (24.8) [23.6-26.1]	1673 (24.4) [23.0-25.9]	
11th	1463 (19.7) [18.6-20.8]	1205 (21.8) [20.2-23.3]	
12th	1545 (21.5) [20.3-22.7]	981 (25.3) [23.5-27.1]	
Race and ethnicity			
Black	3971 (75.1) [73.6-76.6]	2000 (53.1) [51.2-54.9]	
Hispanic	1433 (12.6) [11.8-13.5]	436 (1.1) [0.9-1.3]	
White	618 (12.2) [10.8-13.7]	2138 (45.8) [44.0-47.7]	
Sex			
Male	3524 (48.0) [46.4-49.6]	2750 (48.7) [46.9-50.5]	.26
Female	3507 (52.0) [50.4-53.6]	2768 (51.3) [49.5-53.1]	

^a^ Numbers represent the unweighted sample size of the demographic group. Percentages and 95% CIs are weighted to the respective population of Cleveland and to the first-ring suburbs of Cuyahoga County. Individuals who did not provide a response to grade, race/ethnicity, or sex or did not identify as White, Black, or Hispanic were not included in the summary statistics.

^b^ *P* values are based on χ^2^ tests.

### Youth Tobacco Product Use

Between 2013 and 2015 Cleveland adolescent cigarette use increased from 7.6% (95% CI, 6.7%-8.4%) to 9.1% (95% CI, 8.1%-10.1%) and cigar product use increased from 19.8% (95% CI, 18.5%-21.1%) to 21.3% (95% CI, 20.0%-22.5%), but in the postlegislation period, cigarette use declined to 4.5% (95% CI, 3.9%-5.1%) and cigar product use declined to 16.8% (95% CI, 15.6%-17.9%) (Table 2). Similarly, the prevalence of cigarette use in FRS was increasing in the prelegislation period but declined by more than half in the postlegislation period. Prevalence of cigar product use in FRS was continuously declining. The trends in Cleveland and FRS were different between 2013 and 2017 for cigar products and e-cigarettes, with more notable declines within Cleveland with regard to cigar product use (β = 0.18 [SE, 0.05]; *P* < .001) and more notable declines e-cigarette use within FRS even after controlling for grade and race and ethnicity (β = −0.23 [SE, 0.06]; *P* < .001) (Table 3).

**Table 2.** Prevalence of Tobacco Use Among High School Students in Cleveland City and First-Ring Suburbs of Cuyahoga County, Ohio, Before and After Tobacco 21 Legislation Implementation

Region	Prevalence of tobacco use, No. (%) [95% CI]^a^		
	Prelegislation		Postlegislation, 2017
	2013	2015	
**Cigarettes**			
Cleveland	6562 (7.6) [6.7-8.4]	4874 (9.1) [8.1-10.1]	5895 (4.5) [3.9-5.1]
First-ring suburbs	5322 (10.6) [9.5-11.7]	2312 (11.0) [9.4-12.6]	4040 (5.8) [4.8-6.9]
**Cigar products**			
Cleveland	6201 (19.8) [18.5-21.1]	5877 (21.3) [20.0-22.5]	5784 (16.8) [15.6-17.9]
First-ring suburbs	5163 (16.5) [15.0-18.0]	2697 (15.9) [14.2-17.6]	3943 (14.9) [13.1-16.7]
**e-Cigarettes**			
Cleveland	Not measured	5801 (15.5) [14.4-16.6]	6032 (11.7) [10.8-12.7]
First-ring suburbs	Not measured	2688 (20.1) [18.2-22.0]	4074 (12.4) [10.9-14.0]

^a^ Provided numbers represent the unweighted sample size of the demographic group. Percentages and 95% CIs are weighted to the respective population of Cleveland and to the first-ring suburbs of Cuyahoga County.

**Table 3.** Difference-in-Differences Models Comparing Prevalence of Tobacco Use Among High School Students, Cleveland, Ohio, and the First-Ring Suburbs of Cuyahoga County, Ohio, Before and After Tobacco 21 Legislation Implementation

Assessment	Difference-in-differences model					
	Unadjusted			Adjusted		
	No.^a^	β (SE)^b^	*P* value^c^	No.^a^	β (SE)^b^	*P* value^c^
**By jurisdiction^d^**						
Cigarettes	29 005	−0.02 (0.71)	.76	24 842	0.04 (0.07)	.56
Cigars	29 665	0.15 (0.04)	<.001	25 398	0.18 (0.05)	<.001
e-Cigarettes	18 575	−0.25 (0.05)	<.001	16 140	−0.23 (0.06)	<.001
**Cleveland^e^**						
Cigarettes						
Black						
Male	7665	−0.53 (0.18)	.003	7629	−0.53 (0.18)	.004
Female	7351	0.18 (0.18)	.33	7316	0.19 (0.18)	.30
Hispanic						
Male	3668	−0.23 (0.22)	.29	3642	−0.25 (0.22)	.26
Female	3903	−0.11 (0.22)	.61	3866	−0.04 (0.23)	.86
White						
Male	2233	0.06 (0.21)	.77	2228	0.03 (0.21)	.87
Female	1186	[Reference]	NA	1184	[Reference]	NA
Cigar products						
Black						
Male	12 691	−0.39 (0.07)	<.001	12 627	−0.41 (0.07)	<.001
Female	6175	[Reference]	NA	6138	[Reference]	NA
Hispanic						
Male	8698	−0.33 (0.13)	.010	8641	−0.34 (0.13)	.007
Female	8927	−0.43 (0.13)	<.001	8857	−0.41 (0.13)	.002
White						
Male	7239	0.78 (0.13)	<.001	7202	0.8 (0.13)	<.001
Female	7382	−0.58 (0.13)	<.001	7348	−0.59 (0.13)	<.001
e-Cigarettes						
Black						
Male	5561	0 (0.15)	.99	5531	0.02 (0.15)	.92
Female	5402	0.17 (0.15)	.27	5372	0.19 (0.15)	.20
Hispanic						
Male	2692	0.21 (0.18)	.25	2669	0.23 (0.18)	.21
Female	2874	0.09 (0.18)	.63	2836	0.17 (0.18)	.36
White						
Male	790	[Reference]	NA	787	[Reference]	NA
Female	1669	−0.05 (0.19)	.81	1665	−0.05 (0.19)	.80

Abbreviation: NA, not applicable.

^a^ Provided number represent the unweighted sample size used in the analysis. Each nonreference row for Cleveland represents a model comparing the difference-in-difference between groups and the number is the total number in the model, eg, a total of 2233 White males and White females (reference group) were compared, 1186 of whom were White females.

^b^ Represents the difference-in-difference coefficient or the difference between groups in the pre–Tobacco 21 and post–Tobacco 21 implementation periods.

^c^ Difference-in-difference analysis in this research uses logistic regression to calculate the likelihood of using a select tobacco product use based on exposure to Tobacco 21 legislation. The *P* value presented represents the difference in gains as a result of policy exposure.

^d^ The difference-in-differences models by jurisdiction compares rates in Cleveland to the referent of the first-ring suburbs of Cuyahoga County before Tobacco 21 implementation (2013 and 2015) and after Tobacco 21 implementation (2017). The adjusted models by jurisdiction account for grade level and race and ethnicity.

^e^ The difference-in-differences models in Cleveland compare the rates of demographic groups to the highest prevalence group at baseline for each individual product: White females for cigarettes, Black females for cigars, and White males for e-cigarettes. Models are run pre–Tobacco 21 implementation (2013 and 2015) and post–Tobacco 21 implementation (2017 and 2019). The adjusted models by demographic account for grade level.

Within Cleveland, the prevalence in cigarette use was different for Black male and Black female high school youth compared with all others (Table 4). Trends remained flat, particularly for Black males in the prelegislation period, at 4.5% (95% CI, 3.5%-5.4%) in 2013 and 4.4% (95% CI, 3.2%-5.5%) in 2015. In the same period, prevalence in cigarette use increased particularly for White males, for whom the prevalence increased from 13.7% (95% CI, 9.2%-18.1%) in 2013 to 24.7% (95% CI, 18.2%-31.2%) in 2015. Notably, in the postlegislation period, the prevalence in cigarette use among Black males increased from 2.4% (95% CI, 1.6%-3.1%) in 2017 to 3.6% (95% CI, 2.1%-5.1%), which is the only time product use increased across a demographic group in the postlegislation period. In 2013, the largest disparity was observed between White females (16.1% [95% CI, 11.3%-20.8%]) and Black males (4.5% [95% CI, 3.5%-5.4%]), with a difference of 11.6 (95% CI, 9.5-13.7) percentage points (eFigure 1 in the Supplement). By 2019, the disparity declined by more than half, with the greatest difference being between White females (6.9% [95% CI, 3.4%-10.3%]) and Black females (1.8% [95% CI, 0.7%-2.8%]), with a difference of just 5.1 (95% CI, 3.5-6.7) percentage points.

**Table 4.** Prevalence of Tobacco Use Among High School Students, Cleveland, Ohio, Before and After Tobacco 21 Legislation Implementation

Group	Prevalence of tobacco use, No. (%) [95% CI]^a^			
	Prelegislation		Postlegislation	
	2013	2015	2017	2019
Cigarettes				
Black				
Male	1929 (4.5) [3.5-5.4]	1418 (4.4) [3.2-5.5]	1746 (2.4) [1.6-3.1]	1386 (3.6) [2.1-5.1]
Female	1765 (7.4) [5.9-9.0]	1242 (8.2) [6.5-9.9]	1524 (3.6) [2.5-4.7]	1634 (1.8) [0.7-2.8]
Hispanic				
Male	634 (8.5) [6.1-10.9]	501 (11.1) [7.7-14.5]	679 (6.3) [4-8.7]	668 (3.7) [2.2-5.2]
Female	686 (9.1) [6.7-11.5]	525 (10.2) [7.1-13.2]	692 (6.0) [4.1-8]	814 (2.6) [1.4-3.9]
White				
Male	276 (13.7) [9.2-18.1]	244 (24.7) [18.2-31.2]	252 (11.9) [7.8-16.1]	275 (5.1) [2.2-8.0]
Female	313 (16.1) [11.3-20.8]	308 (16.2) [11.2-21.2]	297 (7.0) [3.8-10.1]	268 (6.9) [3.4-10.4]
**Cigar products**				
Black				
Male	1819 (21.4) [19.4-23.4]	1701 (19.5) [17.5-21.6]	1692 (18.0) [16.0-20.0]	1304 (12.0) [9.6-14.3]
Female	1635 (23.2) [20.7-25.7]	1527 (24.4) [22.1-26.7]	1485 (15.9) [13.9-17.9]	1528 (9.3) [7.3-11.3]
Hispanic				
Male	611 (13.6) [10.5-16.8]	619 (19.3) [15.4-23.1]	661 (14.9) [11.7-18.0]	632 (8.1) [5.8-10.3]
Female	654 (13.9) [10.8-17.0]	646 (15.6) [12.3-18.9]	692 (13.1) [10.1-16.1]	760 (8.7) [6.3-11.1]
White				
Male	270 (7.8) [4.2-11.4]	278 (23.4) [17.6-29.1]	249 (18.4) [13.2-23.6]	267 (12.3) [7.9-16.7]
Female	303 (14.6) [10-19.2]	341 (18.1) [13.2-23.0]	306 (15.3) [10.4-20.2]	257 (12.2) [7.7-16.7]
**e-Cigarettes**				
Black				
Male	Not measured	1685 (10.3) [8.7-11.9]	1769 (7.5) [6.2-8.8]	1317 (5.6) [4.1-7.1]
Female	Not measured	1504 (12.9) [11.1-14.7]	1566 (9.3) [7.7-10.8]	1542 (4.9) [3.4-6.3]
Hispanic				
Male	Not measured	604 (22.8) [18.7-26.9]	695 (15.4) [12.1-18.6]	603 (9.9) [7.3-12.5]
Female	Not measured	637 (23.9) [20-27.8]	715 (18.9) [15.4-22.4]	732 (10.3) [7.7-12.8]
White				
Male	Not measured	277 (26.7) [20.6-32.9]	258 (23.4) [17.4-29.4]	255 (13.2) [8.7-17.7]
Female	Not measured	330 (23.0) [17.3-28.7]	306 (17.4) [12-22.7]	243 (14.4) [9.4-19.4]

^a^ Provided numbers represent the unweighted sample size of the demographic group. Percentages and 95% CIs are weighted to the respective population of Cleveland.

Trends in cigar product use were different among Black and White populations for both males and females compared with all other demographic groups. The greatest prevalence in the prelegislation period was observed among Black males, at 21.4% (95% CI, 19.4%-23.4%), and females, at 23.2% (95% CI, 20.7%-25.7%) in 2013, yet trends were largely flat, particularly for Black males, between 2013 and 2015. All other demographic groups had increases in prevalence, particularly among White males, with an increase from 7.8% (95% CI, 4.2%-11.4%) in 2013 to 24.7% (95% CI, 18.2%-31.2%) in 2015. The largest disparity in 2013 was observed between Black females and White males, with a difference of 15.4 (95% CI, 11.9-18.9) percentage points. The disparity here declined to 4.2 (95% CI, 0.7-7.7) percentage points in 2019, with the greatest prevalence observed among Hispanic males and White males (eFigure 2 in the Supplement).

Rates in e-cigarette use declined similarly across all groups in the prelegislation and postlegislation periods. In 2015, the greatest disparity was observed among White males, for whom prevalence was 26.7% (95% CI, 20.6%-32.9%), and Black males, who had the lowest prevalence, at 10.3% (95% CI, 8.7%-11.9%)—a disparity of 16.4 (95% CI, 13.3-19.5) percentage points. In 2019, the greatest disparity was between White females, at 14.4% (95% CI, 9.4%-19.4%) and Black females, at 4.9% (95% CI, 3.4%-6.3%), or a decrease of 9.5 (95% CI, 7.0-12.0) percentage points (eFigure 3 in the Supplement).

## Discussion

This survey study is the first study, to our knowledge, to examine disparities in adolescent tobacco use within the context of T21 implementation. Cleveland’s T21 policy was associated with reducing use of the most prevalent tobacco product, cigars and cigar-related products, among high school youth in the years following implementation compared with a proximal jurisdiction, as well as reducing disparities in tobacco use across different sex, racial, and ethnic groups.

Students in Cleveland and FRS differed by grade and race and ethnicity, with Cleveland having a higher proportion of students in the 9th grade and a higher proportion of students who were Black or Hispanic. Differences in racial and ethnic makeup likely contributed to differences in the most common products used at baseline, with cigar product use being most prominent in Cleveland and e-cigarette product use most common in FRS. Some studies have found that T21 policies are associated with significantly greater changes among students in younger grades,^20^ with a negligible or opposite outcomes^36^ among older students, which may have contributed to greater declines in Cleveland’s population, yet in our study the difference remained when grade level was controlled for.

There were no observed differences by jurisdiction with respect to the prevalence of cigarette use. While cigarette use has been declining in Cuyahoga County overall since 2011,^37^ this may, in part, be due to youth shifting their use from cigarettes to e-cigarettes, which reflects a broader shift in behavioral norms that has been noted across the US.^38^ While use of e-cigarettes was not captured until 2015, the prevalence of e-cigarette use was greater in both communities compared with cigarette product use. The gap in prevalence of use between cigarettes and e-cigarettes increased from 1.8-fold to 2.1-fold as high in FRS and from 1.7-fold to 2.6-fold as high in Cleveland from 2015 to 2017, further demonstrating a normative shift in tobacco product preferences. Another indicator that highlights this shift are the trends observed immediately following Ohio’s tax increases for cigarettes in July 2015, which would have impacted cigarette prices in both communities.^39^ This policy did not have an immediate association with youth tobacco use trends in either geography, where trends remained relatively stable in FRS and increased slightly in Cleveland before both declining in 2017.

The trajectory for cigarette and little cigar and cigarillo product usage across sex, race, and ethnicity in Cleveland high school–aged youth shifted in the post-T21 implementation period. From 2013 to 2015, the prevalence of current tobacco product use was increasing for all adolescent groups and all products except Black males, for whom use was declining. Immediately after policy implementation in 2017, prevalence dropped across the board for all adolescent populations and continued in 2019 except among Black males who smoked cigarettes, for whom the prevalence increased. A 2016 study by Schneider et al^21^ found that the greatest decline in adolescent tobacco use was immediately after policy implementation and that a longer time horizon may be needed to evaluate whether the policy is associated with shifting the population norm vs immediate changes in trends.

Ongoing surveillance of tobacco retailers in Cleveland conducted by the Prevention Research Center for Healthy Neighborhoods at Case Western Reserve University^40^ suggests that disparities exist in display T21 signage regarding the minimum legal purchasing age, with Black neighborhoods having lower rates of posted signs compared with White or Hispanic neighborhoods. Posting policy-relevant signs helps to foster population norms by promoting awareness by customers, store owners, managers, and employees. A 2021 study by Roberts et al^41^ found that displaying T21 signs was strongly associated with ID checks among retailers.^41^ In Cleveland, this would suggest that the areas with low signage adherence may also be areas with low sales adherence, which may alter the long-term trajectories of youth tobacco use if these disparities persist.

A potential weakness to Cleveland’s T21 policy was the lack of an enforcement plan or strategy for retail violations. Tobacco retail adherence to age-specific sales and ID checking requirements has been mixed. In New York, ID checking adherence declined after T21 implementation,^42^ while California saw low retailer violation rates.^43^ These differences are likely related to the unequal enforcement of T21.^44,45^ A review of local and state T21 policies indicates that very few jurisdictions included enforcement language in their policy.^46^ Having an explicit enforcement component within T21 or subsequent tobacco legislation, such as timelines for adherence inspections or penalty structures for retailer violation, is key to implementation and success.^47^ However, owing to the limitations for state and federal entities to conduct adherence checks, the enforcement responsibility is largely placed on local agencies that lack the necessary resources, which has complicated enforcement.^48^ Furthermore, areas, like Cleveland, with a high prevalence of adult tobacco use likely need enhanced enforcement, given the association between parent and child smoking.^49^ Tobacco retail licensure programs, wherein tobacco retailers pay a fee that is used to fund adherence checks, have emerged as an opportunity for age-specific policy enforcement and have demonstrated effectiveness.^50,51,52^

### Limitations

This study has some limitations. The proximal jurisdictions, the state of Ohio, and the US adopted T21 policies in late 2019, with the exception of 1 city in FRS that implemented T21 in mid-2017. Given that adolescent tobacco use is affected by other factors, such as surrounding communities and use of older peers to purchase the tobacco for them,^53^ adolescents who live closer to Cleveland’s boundaries may still have had access to these peers within the study period prior to more widespread adoption of T21 policies. Broader T21 policy adoption may have also eliminated this access in 2019, further contributing to the observed decline. In addition to the adoption of T21 policies, Ohio imposed an excise tax on vapor products in 2019, which may have contributed to trends observed related to e-cigarettes.

This analysis included high school students some of whom met the minimum age requirements for purchasing tobacco, 18 years, prior to implementation of T21 legislation. This may have contributed to higher use rates prior to legislation. Notably, Cleveland’s T21 policy limits only the sale of tobacco products to those younger than 21 years and does not criminalize tobacco use.

## Conclusions

In this survey study, there was a substantial reduction in tobacco product use among high school youth and a decrease in the magnitude of disparity in tobacco product use across racial, ethnic, and sex demographic groups, demonstrating the potential associated with the T21 policy to achieve equity. Reduction of tobacco product use among high school students and the related tobacco use disparities may subsequently drive down adult tobacco product use and diminish tobacco-related health disparities.

## Notes

Supplement. **eFigure 1.** Prevalence and Disparities in Current Cigarette Use Among High School Students in Cleveland, Ohio, 2013-2019 **eFigure 2.** Prevalence and Disparities in Current Cigar Product Use Among High School Students in Cleveland, Ohio, 2013-2019 **eFigure 3.** Prevalence and Disparities in Current e-Cigarette Use Among High School Students in Cleveland, Ohio, 2013-2019
