# Supplementary material for: Evaluation of Restrictions on Tobacco Sales to Youth Younger Than 21 Years in Cleveland, Ohio, Area
Source: JAMA Netw Open. 2022 Jul 12;5(7):e2222987. doi: 10.1001/jamanetworkopen.2022.22987 (PMC9277498; doi:10.1001/jamanetworkopen.2022.22987)
Supplement: Supplement. — eFigure 1. Prevalence and Disparities in Current Cigarette Use Among High School Students in Cleveland, Ohio, 2013-2019 eFigure 2. Prevalence and Disparities in Current Cigar Product Use Among High School Students in Cleveland, Ohio, 2013-2019 eFigure 3. Prevalence and Disparities in Current e-Cigarette Use Among High School Students in Cleveland, Ohio, 2013-2019 [file jamanetwopen-e2222987-s001.pdf]

Supplemental Online Content

Trapl E, Pike Moore S, Osborn C, et al. Evaluation of restrictions on tobacco sales to youth younger than 21 years in Cleveland, Ohio, area. *JAMA Netw Open*. 2022;5(7):e2222987. doi:10.1001/jamanetworkopen.2022.22987

eFigure 1. Prevalence and Disparities in Current Cigarette Use Among High School Students in Cleveland, Ohio, 2013-2019

eFigure 2. Prevalence and Disparities in Current Cigar Product Use Among High School Students in Cleveland, Ohio, 2013-2019

eFigure 3. Prevalence and Disparities in Current e-Cigarette Use Among High School Students in Cleveland, Ohio, 2013-2019

This supplemental material has been provided by the authors to give readers additional information about their work.

© 2022 Trapl E et al. *JAMA Network Open.*

eFigure 1. Prevalence and Disparities in Current Cigarette Use Among High School Students in Cleveland, Ohio, 2013-2019

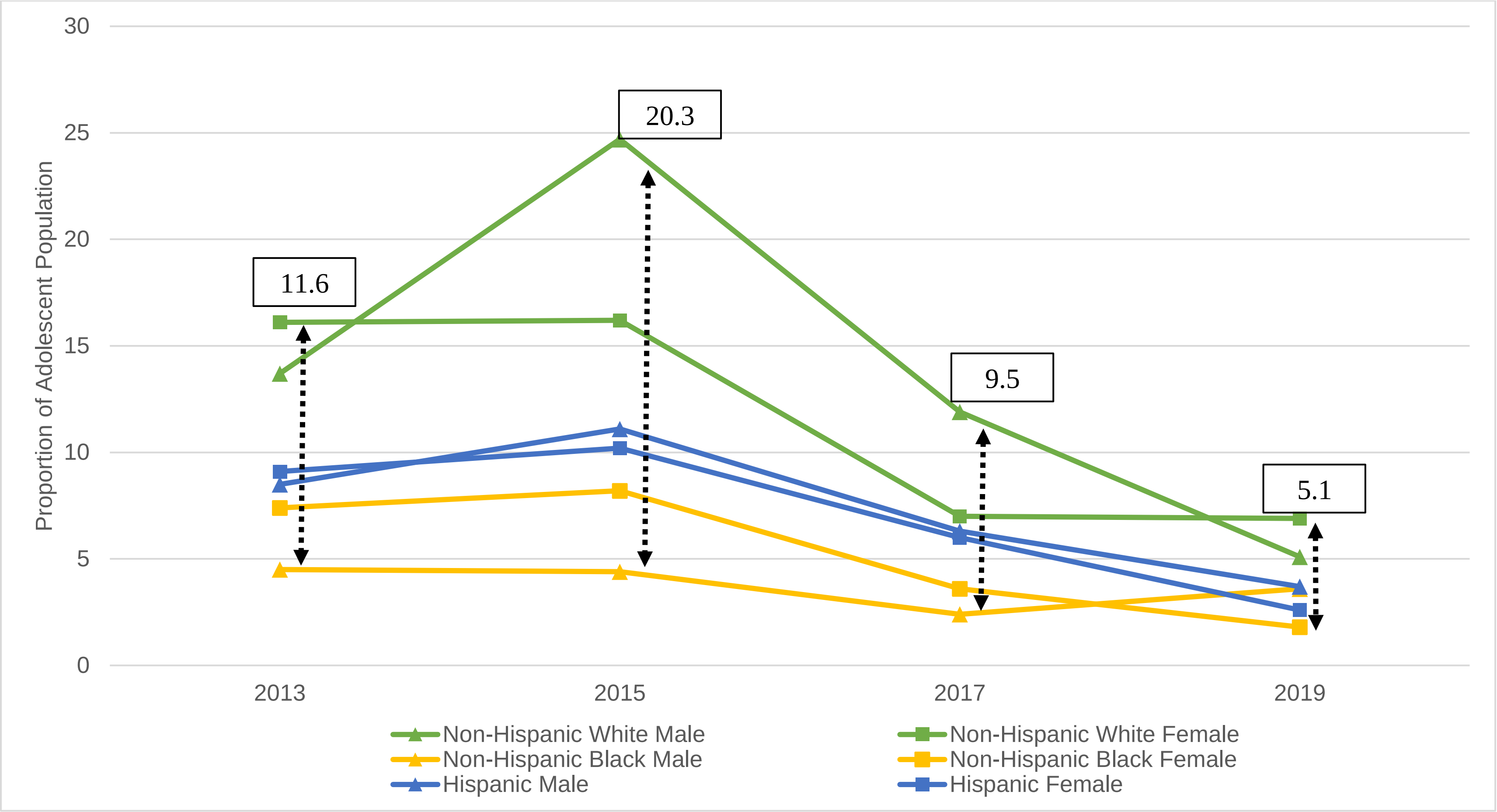

© 2022 Trapl E et al. *JAMA Network Open.*

eFigure 2. Prevalence and Disparities in Current Cigar Product Use Among High School Students in Cleveland, Ohio, 2013-2019

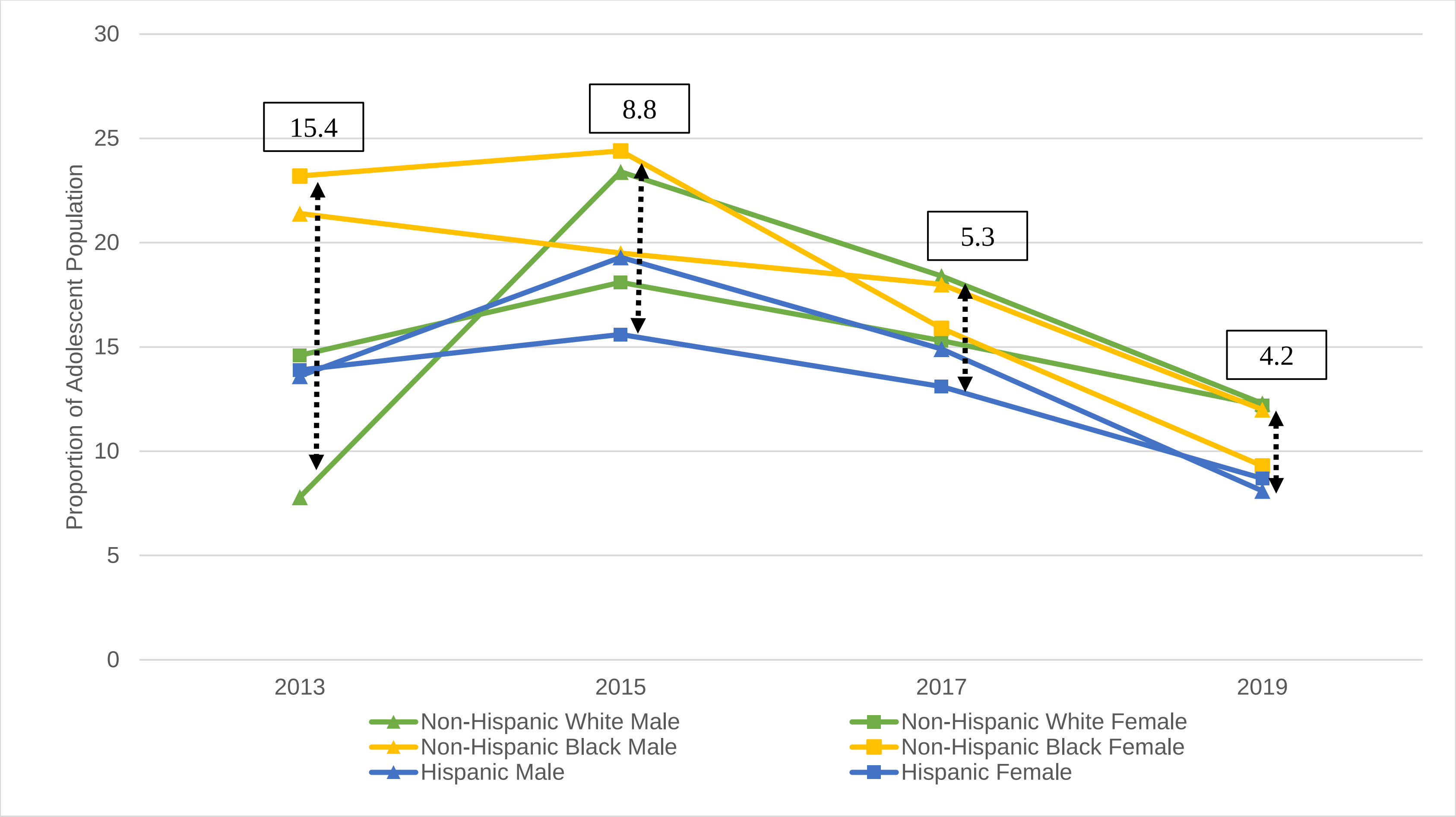

© 2022 Trapl E et al. *JAMA Network Open.*

eFigure 3. Prevalence and Disparities in Current e-Cigarette Use Among High School Students in Cleveland, Ohio, 2013-2019

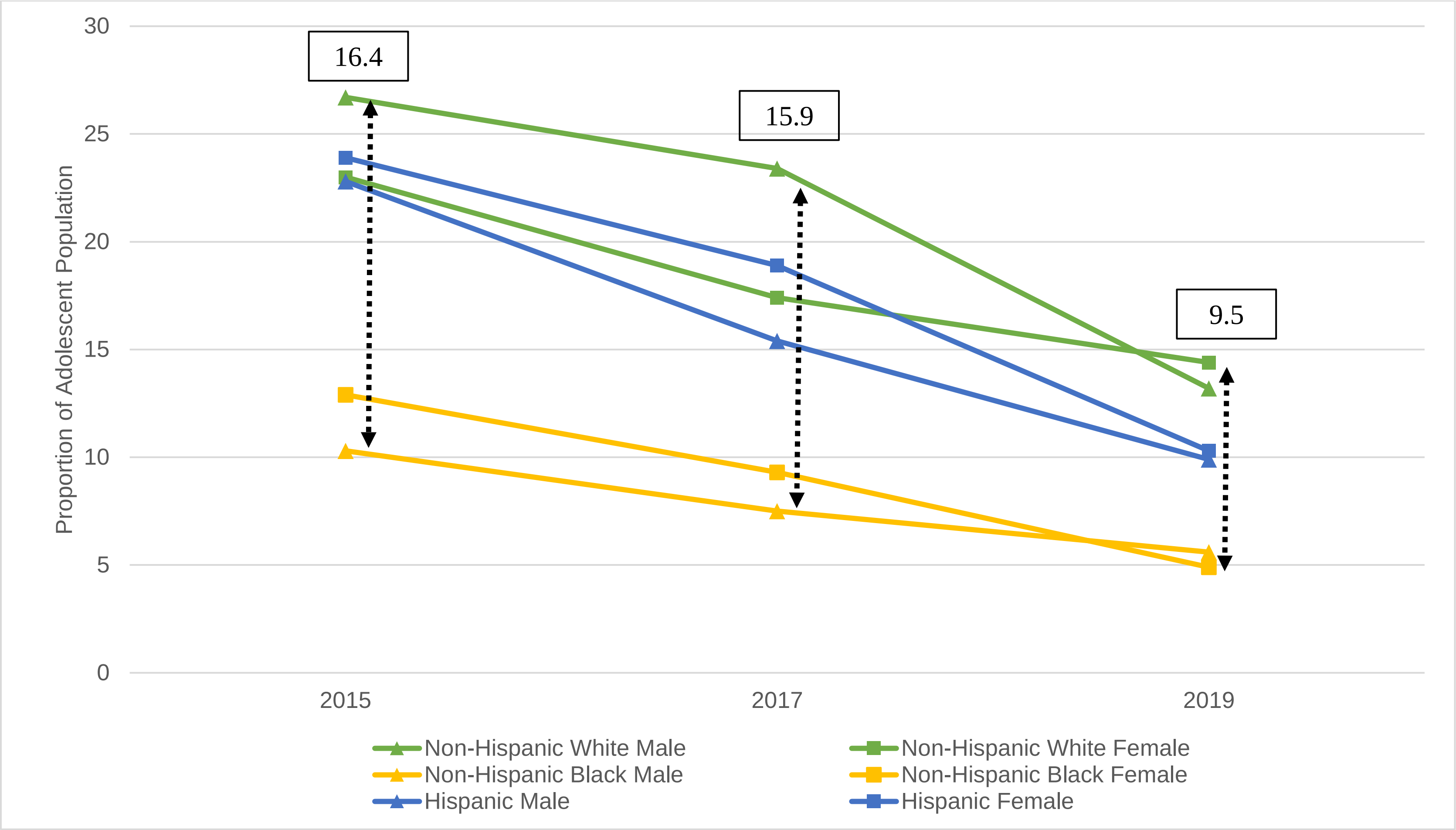

© 2022 Trapl E et al. *JAMA Network Open.*
